# Honey Bees as Diuretic

**Published:** 1858-03

**Authors:** M. B. Beers

**Affiliations:** Portland, Mich.


HONEY BEES AS DIURETIC.

PORTLAND, MICH., Nov. 16th, 1857.

I have used the decoction of the Apis Mellifica or Honey Bee in many cases of suppression of urine, during the last fifteen years, and in almost every case with success. I regard it as far superior to any other diuretic, and have very often given it after other diuretics have failed. I have never tried it in dropsy, and do not know what effect it would have (to give it for some length of time), still I think it would succeed when other means would fail. I have taken from eight to twelve honey bees and poured on them a pint of boiling water, and have given one table-spoonful of the decoction every five minutes; relief will very soon follow. This may not be new to others, but if it is not known, I think it deserves a trial.

I am very respectfully yours, etc.

M. B. BEERS.

*The British and Foreign Medico-Chirurgical Review, or Quarterly Journal of Practical Medicine and Surgery*, for January, 1858, was promptly on our table. Its contents are as varied, interesting and important as in any previous numbers.

*The North-American Medico-Chirurgical Review.* The January number of this very valuable periodical has not reached us.

